# Decreased risk of pneumonia in stroke patients receiving acupuncture: A nationwide matched-pair retrospective cohort study

**DOI:** 10.1371/journal.pone.0196094

**Published:** 2018-05-21

**Authors:** Chuen-Chau Chang, Ta-Liang Chen, Chao-Shun Lin, Chi-Li Chung, Chun-Chieh Yeh, Chaur-Jong Hu, Hsin-Long Lane, Chien-Chang Liao, Chun-Chuan Shih

**Affiliations:** 1 Department of Anesthesiology, Taipei Medical University Hospital, Taipei, Taiwan; 2 Health Policy Research Center, Taipei Medical University Hospital, Taipei, Taiwan; 3 Department of Anesthesiology, School of Medicine, College of Medicine, Taipei Medical University, Taipei, Taiwan; 4 Division of Pulmonary Medicine, Department of Internal Medicine, Taipei Medical University Hospital, Taipei, Taiwan; 5 Department of Surgery, China Medical University Hospital, Taichung, Taiwan; 6 Department of Surgery, University of Illinois, Chicago, IL, United States of America; 7 Department of Neurology, Shuang Ho Hospital, Taipei Medical University, New Taipei City, Taiwan; 8 School of Chinese Medicine for Post-Baccalaureate, College of Medicine, I-Shou University, Kaohsiung, Taiwan; 9 School of Chinese Medicine, College of Chinese Medicine, China Medical University, Taichung, Taiwan; 10 Department of Anesthesiology, Shuan Ho Hospital, Taipei Medical University, Taipei, Taiwan; 11 Ph.D. Program for Clinical Drug Discovery from Botanical Herbs, Taipei Medical University, Taipei, Taiwan; Dartmouth-Hitchcock Medical Center, UNITED STATES

## Abstract

**Background:**

Acupuncture treatment is common among stroke patients, but there is limited information available on whether acupuncture effectively prevents post-stroke pneumonia. The aim of this study was to analyze the differential risk of pneumonia after stroke between patients who did and did not receive acupuncture after discharge.

**Methods:**

We used the Taiwan National Health Insurance Research Database to conduct a retrospective cohort study using propensity score matched-pairs of new stroke patients in 2000–2004 who did and did not receive acupuncture post-stroke. Both cohorts were followed up until the end of 2009 for new-onset pneumonia. After correcting for immortal time bias, the incidence and adjusted hazard ratios (HRs) with 95% confidence intervals (CIs) of pneumonia associated with acupuncture use were calculated using multivariate Cox proportional hazard models.

**Results:**

Overall, 12557 stroke patients with 12557 paired controls were included in the analysis; pneumonia was diagnosed in 6796 (27.1%). Stroke patients receiving acupuncture had a lower incidence of pneumonia than those without acupuncture (53.4 vs. 58.9 per 1000 person-years), with an adjusted HR of 0.86 (95% CI 0.82–0.90). The association between pneumonia risk and acupuncture use was significant in men (HR 0.92, 95% CI 0.86–0.98) and women (HR 0.79, 95% 0.70–0.82) and was also observed in every age group from 20–79 years.

**Conclusion:**

Stroke patients receiving acupuncture had a lower risk of pneumonia than those who did not. Further randomized control studies are needed to validate the protective effect of acupuncture on the risk of pneumonia among stroke patients.

## Introduction

Pneumonia remains the most common serious medical complication among stroke patients and occurs in up to 22% of all stroke cases [1]. Post-stroke pneumonia is known to affect patient outcomes including the occurrence of other complications [2], prolonged hospitalizations, and increased dependency after discharge [1,3]. Pneumonia is also closely related to mortality and highly contributes to the rate of death after stroke [1,4]. In-hospital care for stroke-associated pneumonia in the United States has previously been estimated to cost USD 459 million per year [2], and the recent costs are estimated to be more than twofold higher [3]. Sociodemographic factors including older age, male gender, admission from nursing homes, and preadmission dependency are independent predictors of post-stroke pneumonia [1,4]. Physical status including comorbid chronic obstructive pulmonary disease and coronary artery disease, as well as stroke-related parameters, such as stroke severity, dysphagia, infarct location, impairment of protective reflexes, neurological deficits, and mechanical ventilation, are also known to affect outcomes [1,4,5].

Acupuncture has been accepted as a treatment option and applied in stroke survivors worldwide [6–10]. However, there is limited information on the association between acupuncture and risk of post-stroke pneumonia, and the preventive effects of acupuncture on post-stoke pneumonia remain unclear [11,12]. The purpose of this retrospective cohort study was to assess the protective effect of pneumonia in stroke patients who did and did not receive acupuncture after discharge.

## Methods

### Source of data

Reimbursement claims data from the Taiwan National Health Insurance Program, which was implemented in March 1995 and covers 23 million (> 99%) Taiwan residents, were used in this study. The Taiwan National Health Research Institutes established a National Health Insurance Research Database (NHIRD) that records all beneficiaries' inpatient and outpatient services, including their demographics, primary and secondary diagnoses, procedures, prescriptions and medical expenditures. The validity of NHIRD has been evaluated, and the results have been published globally [6–10].

### Ethical statement

The electronic database was decoded and patient identification data were scrambled to ensure confidentiality; informed consent was thus exempted. We conducted this study in accordance with the Declaration of Helsinki. This study was also evaluated and approved by the Institutional Review Board of E-DA Hospital, Kaohsiung, Taiwan (EDA-JIRB-2014012; EDA-JIRB-2017004).

### Study design

We used the same dataset from the NHIRD in our previous studies [6–10]. Briefly, of the 23 million people in Taiwan, 226,699 patients aged ≥20 years with newly diagnosed stroke who were admitted to the hospital between Jan 1, 2000, and Dec 31, 2004, were considered eligible subjects. To identify eligible stroke patients and pneumonia events, the following exclusion criteria were applied: (1) patients who died during the index stroke admission; (2) patients who stayed in the hospital for ≥30 days during the index stroke admission; and (3) patients who had been diagnosed with pneumonia within 6 months prior to the index stroke admission. The diagnosis of stroke was validated as described in previous studies [6–10]. In total, 12557 stroke survivors received at least two courses (one course consisted of six consecutive treatments) of acupuncture treatment during the follow-up period until the end of 2009; these patients were defined as the acupuncture group (those with only one course of acupuncture were excluded).

Stroke patients without acupuncture were selected using a matched pair procedure with propensity scores (exposure vs. non-exposure ratio = 1:1). For the non-acupuncture group, stroke patients were followed up from the date of discharge after stroke admission (index date) until December 31, 2009, a pneumonia event, loss to follow-up, or death. For the acupuncture group, stroke patients were followed up from the first date of acupuncture treatment after the stroke admission (index date) until December 31, 2009, a pneumonia event, loss to follow-up, or death. In the acupuncture group, the time period between the discharge date and the date of first acupuncture treatment after stroke admission represented the immortal time. Therefore, immortal time bias (resulting from an overestimation of the intervention’s beneficial effects) was avoided in this study. Follow-up time, in person-years, was calculated for each stroke patient from the index date to the end point. We compared the risk of pneumonia between the matched pairs of stroke patients and the controls during the follow-up period.

### Criteria and definitions

We defined newly diagnosed stroke by the *International Classification of Diseases*, *9th Revision*, *Clinical Modification* codes (ICD-9-CM 430–437). Subtypes of stroke were further classified as hemorrhagic (ICD-9-CM 430–432), ischemic (ICD-9-CM 433, 434), and other (ICD-9-CM 435–437). Pneumonia, the primary outcome, was defined by ICD-9-CM codes 480–486. Low-income status was defined as meeting the condition to waive medical copayments, as determined by the Bureau of National Health Insurance, Taiwan. We calculated the population density by dividing the population by the area of each of the 359 townships and city districts in Taiwan. Urbanization was defined as low (first quartile of population density), moderate (second and third quartiles), and high (fourth quartile). Coexisting medical conditions were determined from medical claims data during the follow-up period and included diabetes (ICD-9-CM 250), hypertension (ICD-9-CM 401–405), hyperlipidemia (ICD-9-CM 272.0, 272.1, and 272.2), mental disorders (ICD-9-CM 290–319), liver cirrhosis (ICD-9-CM 571.2, 571.5, 571.6), ischemic heart disease (ICD-9-CM 410–414), urinary tract infection (ICD-9-CM 599.0), and heart failure (ICD-9-CM 428), dysphagia (ICD-9-CM 787.2, 438.82). Admission to the intensive care unit, neurosurgery during the index hospitalization, and length of hospital stay were also identified as potential confounders.

### Statistical analysis

We estimated the propensity scores for acupuncture use by a non-parsimonious logistic model, considering all significant clinical covariates proposed. We applied a structured iterative approach to refine this model and achieve a balance of covariates within matched pairs, using a greedy matching strategy with the nearest-neighbor algorithm. This method has been reported to remove 98% of the bias from measured covariates [13]. Chi-square tests and t tests were used to measure the distribution of covariates between stroke patients with and without acupuncture use, and a p-value <0.05 indicated a meaningful difference.

We then utilized multivariate Cox proportional hazard models to control for confounders and calculated adjusted hazard ratios (HRs) and 95% confidence intervals (CIs) for pneumonia in terms of acupuncture use. We performed further subgroup analyses for each stratum of sex, age group, and subtype of stroke using the full model without the stratifying variable. The number of sessions of acupuncture use was also considered an independent factor associated with post-stroke pneumonia in the multivariate Cox proportional hazard model. Kaplan-Meier analysis was used to calculate the cumulative probability of pneumonia in stroke patients with and without acupuncture use, and the group differences between the cumulative incidence curves were evaluated by log-rank test. All analyses were performed using Statistical Analysis Software version 9.1 (SAS Institute Inc., Cary, NC, USA). A two-sided p value <0.05 was considered statistically significant.

## Results

After propensity score matching (Table 1), there were no significant differences between groups in age, sex, stroke subtype, income, urbanization, diabetes, hypertension, hyperlipidemia, mental disorder, liver cirrhosis, ischemic heart disease, urinary tract infection, heart failure, COPD, epilepsy, renal dialysis, stay in intensive care unit, neurosurgery, hospitalized rehabilitation, type of hospital, cardiovascular medications, or length of hospital stay.

**Table 1:** The characteristics of hospitalized stroke patients.

	No use		Acupuncture use		
	N = 12557		N = 12557		p-value
Sex	n	(%)	n	(%)	1.0000
Female	5297	(42.2)	5297	(42.2)	
Male	7260	(57.8)	7260	(57.8)	
Age at first stroke, years					1.0000
20–29	74	(0.6)	74	(0.6)	
30–39	183	(1.5)	183	(1.5)	
40–49	1168	(9.3)	1168	(9.3)	
50–59	2546	(20.3)	2546	(20.3)	
60–69	4294	(34.2)	4294	(34.2)	
70–79	3688	(29.4)	3688	(29.4)	
≥80	604	(4.8)	604	(4.8)	
Low income	74	(0.6)	74	(0.6)	1.0000
Urbanization					1.0000
Low	253	(2.0)	253	(2.0)	
Moderate	4240	(33.8)	4240	(33.8)	
High	8064	(64.2)	8064	(64.2)	
Type of hospital					1.0000
Medical center	2473	(19.7)	2473	(19.7)	
Regional hospital	4842	(38.6)	4842	(38.6)	
Distinct hospital	5242	(41.8)	5242	(41.8)	
Subtypes of stroke					1.0000
Hemorrhage	859	(6.8)	859	(6.8)	
Ischemic	7292	(58.1)	7292	(58.1)	
Others	4406	(35.1)	4406	(35.1)	
Coexisting medical condition					
Diabetes Mellitus	4122	(32.8)	4122	(32.8)	1.0000
Hypertension	9044	(72.0)	9044	(72.0)	1.0000
Hyperlipidemia	1591	(12.7)	1591	(12.7)	1.0000
Mental disorder	4054	(32.3)	4054	(32.3)	1.0000
Liver cirrhosis	29	(0.2)	29	(0.2)	1.0000
Ischemic heart disease	3027	(24.1)	3027	(24.1)	1.0000
UTI	2078	(16.6)	2078	(16.6)	1.0000
Congestive heart failure	264	(2.1)	264	(2.1)	1.0000
COPD	3207	(25.5)	3207	(25.5)	1.0000
Epilepsy	46	(0.4)	46	(0.4)	1.0000
Dysphagia	29	(0.2)	29	(0.2)	1.0000
Dysphagia past two years	28	(0.2)	28	(0.2)	1.0000
Renal dialysis	43	(0.3)	43	(0.3)	1.0000
ICU stay in stroke admission	586	(4.7)	586	(4.7)	1.0000
Suctioning in stroke admission	373	(3.0)	277	(2.2)	0.0001
Bacterial sensitivity test in stroke admission	589	(4.7)	420	(3.3)	< .0001
General ward stay in stroke admission	12339	(98.3)	12381	(98.6)	0.0329
Nasogastric intubation in stroke admission	832	(6.6)	609	(4.9)	< .0001
Osmotherapy in stroke admission	1625	(12.9)	1679	(13.4)	0.3134
Urinary catheterization in stroke admission	957	(7.6)	801	(6.4)	0.0001
Neurosurgery in stroke admission	150	(1.2)	150	(1.2)	1.0000
Rehabilitation in stroke admission	3232	(25.7)	3232	(25.7)	1.0000
Anticoagulants	522	(4.2)	522	(4.2)	1.0000
Anti-platelet agents	11821	(94.1)	11821	(94.1)	1.0000
Lipid-lowering agents	6240	(49.7)	6240	(49.7)	1.0000
Length of stay, Mean±SD	6.20±4.65		6.21±4.68		0.9288

**Fig 1:**
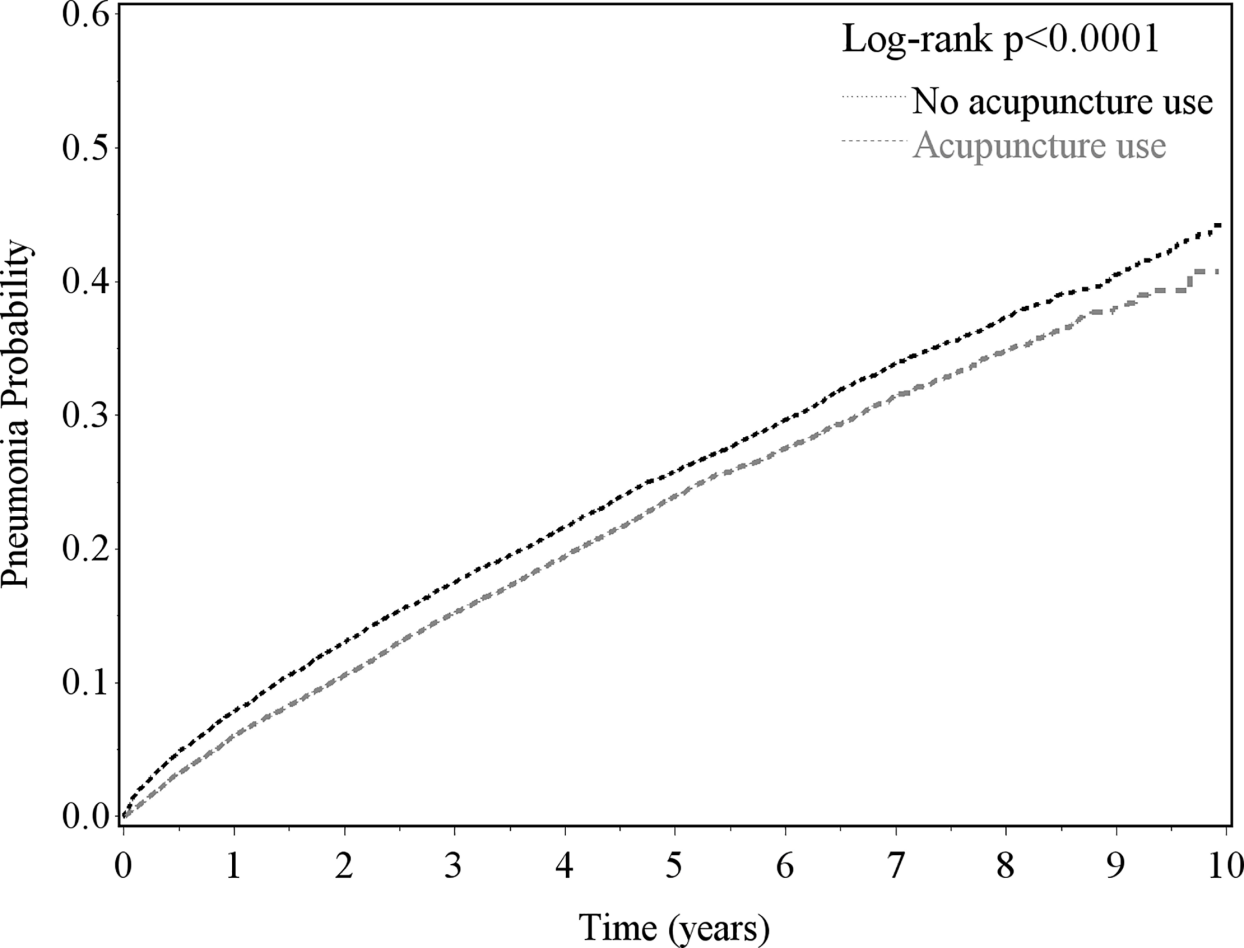
Kaplan-Meier analysis for pneumonia risk in new-diagnosed stroke patients in 2000–2004 with and without acupuncture use for follow-up to the end of 2009.

During the follow-up period (Table 2), the acupuncture group had a lower incidence of pneumonia than the control group (53.4 vs. 58.9 per 1000 person-years), with an adjusted HR of 0.86 (95% CI 0.82–0.90). The log-rank test (Fig 1) indicated that the acupuncture group had a lower probability of pneumonia than the control group (p <0.0001).

**Table 2:** Incidence and adjusted hazard measures of pneumonia in association with acupuncture use by sex, age at first stroke and subtypes of stroke.

	No use			Acupuncture use			Pneumonia risk	
	Events	PY	IR	Events	PY	IR	HR	(95% CI)*
Full model	3825	64904	58.9	2971	55596	53.4	0.86	(0.82–0.90)
Sex								
Female	1473	28623	51.5	1009	24103	41.9	0.79	(0.70–0.82)
Male	2352	36281	64.8	1962	31493	62.3	0.92	(0.86–0.98)
Age, years								
20–29	13	387	33.6	2	366	5.5	0.16	(0.04–0.69)
30–39	31	956	32.4	15	839	17.9	0.52	(0.28–0.98)
40–49	171	6839	25.0	112	5657	19.8	0.73	(0.58–0.93)
50–59	486	14836	32.8	362	12364	29.3	0.83	(0.72–0.95)
60–69	1254	23222	54.0	920	19532	47.1	0.82	(0.75–0.89)
70–79	1553	16896	91.9	1257	14952	84.1	0.88	(0.81–0.95)
≥80	317	1769	179.2	303	1885	160.7	0.86	(0.73–1.00)
Subtypes of stroke								
Hemorrhagic	215	4310	49.9	147	4008	36.7	0.66	(0.53–0.81)
Ischemic	2324	36768	63.2	1862	32254	57.7	0.86	(0.81–0.92)
Others	1286	23826	54.0	962	19334	49.8	0.87	(0.80–0.95)
Medical conditions								
0	471	4285	109.9	307	5115	60.0	0.49	(0.43–0.57)
1	971	14550	66.7	717	13006	55.1	0.77	(0.70–0.85)
2	1021	17711	57.6	816	14964	54.5	0.92	(0.84–1.01)
≥3	1362	28539	48.0	1131	22511	50.2	1.10	(1.01–1.19)
Length of stay, days								
1–6	2420	44568	54.3	1820	36260	50.2	0.90	(0.84–0.95)
≥7	1405	20336	69.1	1151	19336	59.5	0.80	(0.74–0.87)

*Adjusted for all covariates listed in Table 1. The HR of pneumonia associated with acupuncture use is 0.86 (95% CI 0.82–0.91) in the additional full model adjusted all covariates listed in Table 1 plus suctioning, bacterial sensitivity test, general ward stay, nasogastric intubation, osmotherapy and urinary catheterization.

†In the sensitivity analysis, acupuncture was associated with reduced pneumonia risk after excluding pneumonia event in the 1 (OR 0.87, 95% CI 0.83–0.91), 3 (OR 0.88, 95% CI 0.84–0.93), 6 (OR 0.90, 95% CI 0.86–0.94), 9 (OR 0.91, 95% CI 0.86–0.95), 12 (OR 0.92, 95% CI 0.87–0.96) months during the follow-up period.

In subgroup analyses, the beneficial effects of acupuncture on pneumonia risk remained significant in men (HR 0.92, 95% CI 0.86–0.98) and women (HR 0.79, 95% 0.70–0.82), with a tendency toward favoring the female gender. The adjusted HRs were significant in all age strata, ranging from 0.16 (95% CI 0.04–0.69) in those aged 20–29 years to 0.88 (95% CI 0.81–0.95) in patients 70–79 years old. The association between acupuncture treatment and reduced pneumonia risk was also significant for hemorrhagic stroke (HR 0.66, 95% CI 0.53–0.81), ischemic stroke (HR 0.86, 95% CI 0.81–0.92), and other stroke (HR 0.87, 95% CI 0. 0.80–0.95). The adjusted HRs of pneumonia risk associated with acupuncture treatment for stroke patients with 0 and 1 medical condition were 0.49 (95% CI 0.43–0.57) and 0.77 (95% CI 0.70–0.85), respectively. Regardless of whether patients’ index stroke admission was 1–6 (HR 0.90, 95% CI 0.84–0.95) or ≥7 (HR 0.80, 95% CI 0.74–0.87) days, use of acupuncture was associated with reduced pneumonia risk.

Moreover, acupuncture treatment showed a significant frequency-dependent association with reduced pneumonia in stroke patients receiving 4 (HR 0.88, 95% CI 0.78–0.99), 5 (HR 0.84, 95% CI 0.73–0.96), and ≥6 continuous sessions (HR 0.77, 95% CI 0.73–0.82) of acupuncture therapy (Table 3).

**Table 3:** Numbers of package of acupuncture use in association with post-stroke pneumonia risk.

Numbers of package	n	Events	Person-year	Incidence	HR	(95% CI)*
0	12557	3825	64904	58.9	1.00	(reference)
2	2740	645	10385	62.1	0.98	(0.91–1.07)
3	1801	451	7326	61.6	0.99	(0.90–1.09)
4	1236	299	5287	56.6	0.88	(0.78–0.99)
5	918	212	4106	51.6	0.84	(0.73–0.96)
≥6	5862	1364	28493	47.9	0.77	(0.73–0.82)

*Adjusted for all covariates listed in Table 1.

## Discussion

Using data from the NHIRD, we found a reduced risk of pneumonia in newly diagnosed stroke patients when they received acupuncture after discharge in this nationwide, propensity score-matched, retrospective cohort study. Our analyses revealed that the reduction in risk tend to be most significant in women, younger patients, and patients with hemorrhagic stroke.

Studies focusing on the preventive effects of acupuncture on post-stroke pneumonia are relatively rare [12], and the conclusions have been inconclusive due to small sample sizes and methodological deficiencies [11]. We previously examined the effects of in-hospital traditional Chinese medicine (TCM) on preventing post-stroke complications within 6 months after discharge from stroke hospitalization and found a significant reduction in pneumonia by adjuvant TCM therapy, 96% of which consist of acupuncture [6]. In this study, we further focused on the correlation between acupuncture and post-stroke pneumonia and extended the follow-up period in a significantly larger cohort. Interestingly, the tendency toward stronger effects in women, younger groups, and non-hemorrhagic stroke patients was also noted in our previous research on post-stroke acute myocardial infarction and epilepsy [8, 9].

Several factors might explain the mechanisms behind our findings. First, acupuncture may increase regional cerebral blood flow [14–16], which may exert some neuroprotective effects [9,17]. Increased cerebral blood flow by acupuncture has been associated with enhanced perfusion mediated by endothelial nitric oxide synthase [18], attenuated angiotensin II activity [19], and angiogenesis promotion in animal models [20] and with improved carbon dioxide reactivity [16], decreased sympathetic activity [21], and increased endothelial progenitor cell count and functioning in human studies [22]. The hypothesized biophysical mechanisms of the neuroprotective effects include angiogenesis promotion, alleviation of inflammatory responses, regulation of the blood brain barrier, and inhibition of apoptosis [23].

Second, dysphagia has been considered a risk factor for post-stroke pneumonia and subsequent mortality [24]. The beneficial effects of acupuncture on improving dysphagia have been investigated in previous studies [12, 25, 26]. Based on the results of our study and of previous related reports, we postulate that acupuncture might help decrease post-stroke pneumonia through its beneficial effects on dysphagia.

Third, the suppression of immune function after stroke should be considered in light of three aspects. First, central nervous system injuries, including stroke, are known to reduce and disrupt peripheral immune cells and suppress their functions [27, 28]. Additionally, stroke is associated with various common and persistent painful conditions [29, 30]. Most patients’ pain has moderate or severe effects on their daily lives and remains untreated or becomes treatment-resistant [30]. However, we have no related data to show the effects of acupuncture on post-stroke pain and it needs further researches [31]. Finally, stroke survivors commonly experience varying degrees of mood disorders including depression and anxiety [32, 33]. Although the prevalence of these conditions fluctuates over time [34], post-stroke emotional stresses remain a common problem several years after the event [35]. Chronic pain syndromes and emotional stresses are known to suppress immune function including decreased peripheral natural killer cell count and activities and increased metastasis of tumor cells [36]. Acupuncture can directly regulate specific [37] and nonspecific immunity [38]. Additionally, it has long been used, and proven effective, to cure chronic pains [39] and emotional disorders [40]. We postulate that acupuncture might correct for the immunosuppression that occurs after stroke either directly, by modulating the specific and non-specific immunity, or indirectly, by ameliorating post-stroke pain and emotions, thereby decreasing the incidence of post-stroke pneumonia.

The strengths of this NHIRD-based study include the large sample size, long follow-up period, and minimized selection bias. Second, we applied propensity score matching to minimize residual influences from socio-demographic factors and coexisting medical conditions, and we also used multivariate Cox proportional hazard models to control for confounding effects. Third, to reduce the immortal time bias, we corrected for immortal time in the acupuncture group when calculating person-years. Finally, we performed subgroup analyses to test the robustness of our estimates and demonstrated the trends in differential effects across the tested strata.

Some limitations of this study should be addressed. First, we used insurance claims data without detailed information on clinical risk scores, lesion characteristics, biochemical measures, or patient lifestyles. Second, information on the actual acupuncture points and manipulations applied in treatment was not available. Inadequately standardized management protocols might have affected the accuracy of the estimated treatment effects and should be considered a study limitation. Third, although the accuracy of diagnostic codes in the database has been verified in previous studies [6–10], the validity of comorbidity codes is another potential limitation. In addition, we cannot infer causality regarding the association between acupuncture and post-stroke pneumonia because our study was observational. Finally, although we used a propensity score matching procedure and multivariate regression models to adjust for the covariates in stroke patients with and without acupuncture, residual confounding remains a possibility.

In conclusion, we showed that stroke patients receiving acupuncture had a reduced risk of pneumonia in this nationwide retrospective cohort study. The potential beneficial effects of acupuncture as a supplemental measure to reduce the occurrence of pneumonia deserve further exploration in well-designed controlled clinical trials with stringent acupuncture protocols and blinded sham control designs.
